# Supplementary material for: MetaRibo-Seq measures translation in microbiomes
Source: Nat Commun. 2020 Jun 29;11:3268. doi: 10.1038/s41467-020-17081-z (PMC7324362; doi:10.1038/s41467-020-17081-z)
Supplement: Supplementary file 10 — Supplementary Data 7 [file 41467_2020_17081_MOESM10_ESM.zip › File2/Confidence_VeryHigh_Taxonomy/402704_out.krona.html]

Javascript must be enabled to view this page.

members
magnitude
magnitudeUnassigned
count
unassigned
taxon
rank

402704\_out

10

2
10
superkingdom

phylum
10
1239

909932
class
1

1843488
1
order

909930
1
family

904
genus
1


SRS053214\_contig\_number\_21346
1203555
species
1

class
9
186801

186802
9
order

2
family
186803

species
1

SRS024009\_contig\_number\_9054
658087

genus
1
207244

species
1
649756

SRS142503\_contig\_number\_contig-100\_5303.288465

541000
family
4

1
genus
1263


SRS022137\_contig\_number\_6454
165186
species
1

946234
3
genus

1193534

SRS020622\_contig\_number\_3056SRS078665\_contig\_number\_contig-100\_210.77043SRS143876\_contig\_number\_9637
3
species

1950905

SRS011405\_contig\_number\_8110SRS054352\_contig\_number\_20137
species
2

2109688

SRS148511\_contig\_number\_4068
species
1
